# On the Use of Nitric Acid in Hæmorrhoids

**Published:** 1855-01

**Authors:** Henry Smith


On the use of Nitric Acid in Hæmorrhoids. By Henry Smith, F. R. C. S.—Several years have now elapsed since the attention of surgeons was directed by Dr. Houston to the improved treatment of certain forms of piles by the application of nitric acid, and since the introduction of this plan, it has doubtless been employed by many in the profession; but it seems to be necessary even now to direct further attention to this matter. Most of us may be acquainted with the fact of a certain improved mode of treatment, or a certain remedy having been recommended and brought into the category of surgical means and appliances; but this may be all; for many of us either have no faith in it, or are too much wedded to the use of those other methods which have been adopted by our teachers and by the previous generations of Surgeons.

These remarks may, I believe, be truly applied to the subject under present notice. The great advantages of nitric acid in the treatment of some forms of hæmorrhoidal tumours are not yet sufficiently known and acknowledged; and, having had numerous opportunities of observing them, I deem it right to give the results of some of this experience, and to make some observations thereon.

In many cases of hæmorrhoids, the distress caused by them, perhaps for years, has been such, that many patients are induced to undergo operations of considerable severity for the purpose of getting cured. Ligature of the offending part and excision have been the measures to which surgeons have for the most part resorted.

When the hæmorrhoids are situated within the sphincter ani, excision is a dangerous remedy. It is true that in many cases the scissors may be used without much bleeding, and a good cure will result; but every now and then such extensive hæmorrhage will follow as to place the life of the patient in jeopardy. I have myself been called to two such instances. In the one case of a young-gentleman, some internal piles had been cut off by another surgeon. Three hours after the operation I was called to the patient, and found that he had bled enormously; in truth, his life had been seriously perilled.

In the second case, the patient was a middle-aged female, on whom I had performed a somewhat complicated operation for the relief of a prolapsed rectum, conjoined with excessively painful piles. I snipped off the most prominent part of the swelling which was protruding externally, and after having returned the parts within the sphincter, and perceiving no bleeding, left the patient, but in about a quarter of an hour she suddenly had an inclination to stool, and passed an immense quantity of blood. This occurred two or three times, and when I saw her she was in a very exhausted and restless state; but I managed to stop the bleeding, and she happily recovered. In this instance I doubtless used the scissors more freely than was proper.

It may be stated as a rule, with but few exceptions, that the scissors or knife should be limited to those excrescences, vascular or otherwise, which are situated external to the sphincter; where there is a prominent swelling, with a more or less narrow neck growing from the verge of the anus; where there is simply an enlarged and distended vein containing a coagulum of blood; and especially where, with a relaxed and hæmorrhoidal condition of the rectum, there is a redundancy of loose skin around the anus—in such latter case the propriety of using the scissors is very obvious.

Ligature of internal piles or of portions of a relaxed state of the rectum is an operation which is chiefly in favor among surgeons, and its use is attended with most signal benefit; and, if it were invariably adopted without producing dangerous or fatal symptoms, it would not be expedient to discard the employment of such a remedy; for, in general, but one operation is required, and, if this be properly effected, the benefit will be speedy and lasting; but the advantages of the ligature are, to a certain extent, counterbalanced by certain evil results which are likely to follow its use. In the first place, the patient must be confined to his bed or sofa for some days. Secondly, the application of the ligature is sometimes followed by the most intense pain. Thirdly, symptoms of an alarming and dangerous character may be produced; and, fourthly, death itself may, and does occasionally follows its use.

I have had no personal experience of a fatal event after the use of the ligature; but I know of one instance in the practice of a friend where death did ensue after an operation, and, during a recent discussion at the London Medical Society, Mr. Henry Lee, who has paid much attention to this subject, stated that he had examined two individuals where death had been produced by the ligature. In one instance of a gentleman—a most valuable life—who had had a ligature applied for the cure of a bad prolapsus, most alarming peritoneal symptoms came on, so that I was obliged to watch him most carefully for some time after the operation; and in others I have seen very severe pain and distress produced,—retention of urine is by no means an uncommon sequela.

Under these circumstances it is desirable to employ an agent which may effect the same good purpose without the attendant evil results; and, in many instances, this may obtain in the use of nitric acid. It would be absurd to attempt to discard the use of the ligature in all; for, where there is considerable prolapsus of the rectum, or where the hæmorrhoidal tumours are large and have extensive bases, it will be necessary to resort to the use of the ligature; but, in a great majority of instances of internal piles and of prolapsed condition of the rectum dependent upon the unhealthy and vascular state of the mucous membrane, nitric acid will be found to effect a cure; and this, too, without causing any of those painful and distressing symptoms which occasionally follow the use of the ligature. It has occurred to me to have many opportunities of employing it, and I have seen most excellent results from it. I will briefly relate a few of the most striking cases.

In May last, I was requested to see Mr. F., a gentleman who had just returned from Australia, in company with his medical attendant, who told me that he had suffered for several years with distressing protrusion of the rectum after going to stool, and that he was most anxious to get cured, and would undergo any operation.

On examination, I discovered that there was a highly congested and relaxed state of the whole mucous membrane of the rectum, and just within the sphincter a vascular broad mass, which might or might not have been termed a pile. However, the pain after going to stool was always most excessive as the mucous membrane prolapsed. In addition, there was an external hæmorrhoidal excrescence attached to the verge of the anus. I consulted with the surgeon in attendance, who had not even heard of the use of nitric acid before he came to England, and it was determined to apply it freely to the whole congested and relaxed portions of the rectum.

The patient was very anxious that only one operation should be done; therefore the very strongest acid was applied with freedom, and the external pile was cut off.

It was not necessary to apply it again. Great pain was produced by the acid, lasting some time, but when his bowels were first moved after the operation there was not any prolapsus, and in a few days he had lost all trace of a complaint which had for years embittered his life.

While this patient was under my care, Mr. T., a gentleman aged 65, consulted me about his piles. He had suffered for more than twenty years, and had consulted various practitioners, and had used a quantity of remedies. The last person he consulted was an homœopath, who took the fees of the old man with an unsparing hand, but did not even condescend to examine the rectum. He stated that his sufferings had been great for the last twenty years; that the gut came down when he went to stool, which process lasted an indefinite period of time, in consequence of his being compelled to return the protruded part, which at times he assured me had sometimes filled the hollow of his hand. In addition to this, the gut generally prolapsed when he went out for a walk, and at times the hæmorrhage had been such, that, after leaving his house for a walk, he had been compelled to return hastily to change his drawers.

On examination, I found a collection of very vascular and strawberry-colored piles within the sphincter, and a protrusion of the mucous membrane of the rectum. Around the anus was a large quantity of relaxed and thickened integument. It was just the case which was adapted for the treatment by nitric acid, the action of which I explained to the patient, who readily consented to its use.

On the following day, his bowels having been cleared out, and the mucous membrane being protruded as much as possible by the use of warm vapour, I applied nitric acid lightly to the largest pile. The effect of this was only to give pain for a few moments, and, after a few days, there was decided relief, when the acid was repeated to others of the tumours. The same treatment was carried on until I had applied it on five different occasions, when, with scarcely giving the patient any amount of pain, all the excrescences were nearly destroyed. The loose and redundant skin around the anus was then removed, and in a few days the patient came to me full of gratitude, stating that he had no further trouble, and that he had not been in such comfort for twenty years.

In the first of these cases the cure was more rapid and decided than I could have expected, as it was not one for which the nitric acid treatment is so well adapted, the disease consisting mainly of a highly congested state of the veins of the rectum, the hæmorrhoidal tumour itself being of a deep blue color. It is in the instances like the second, where the tumours are defined, very vascular, and of a bright red color resembling a strawberry, that the nitric acid is so particularly useful.

But it is in cases where there is a great deal of bleeding that it acts so well; in the second there had been most copious hæmorrhage from time to time, but this had been checked before the patient consulted me, and therefore it would not be fair to give the acid the credit of curing this as well as other symptoms, because it did not exist at that precise period of time; but cases have occurred to me in which I have seen remarkable effects produced as regards arrest of bleeding. The last case treated by me was an instance of this. It occurred only a few weeks ago, in the person of an elderly woman, who came to me complaining of profuse bleeding from the rectum, which had been going on for several days. Her countenance betokened loss of blood. On examination, I discovered, just within the sphincter, a large, vascular, bright red pile. I touched it lightly with nitric acid; this had the effect of restraining the hæmorrhage, and after three other applications she was cured.

If the remedy is thoroughly applied, and the accessory treatment properly pursued, the cure will be lasting, a striking instance of which was presented to my notice a few weeks ago, in the person of an officer in the Navy, who was under my care in 1850 for the treatment of very bad piles, from which he had suffered greatly many years, he having been employed on active service in a hot climate. Here there were several piles internally, and a large redundancy of loose skin externally. Nitric acid was applied three or four times to the former, and the loose skin was cut away; a good cure resulted. I saw this gentleman the other day on his return from active service abroad, and he tells me he is quite well.

In many instances of long standing hæmorrhoids, the external skin becomes so thickened and relaxed that there will be a necssity of snipping it all away if a perfect cure be expected. The contraction which takes place subsequently to the operation braces up the parts and prevents any further protrusion of the gut which might subsequently take place.

The acid should be quite pure, and should be applied by means of a stick of wood. The parts should be well oiled afterwards. It is very important to attend to one thing, namely, to wipe the part to which the acid is to be applied carefully with a piece of lint, in order to get rid of the mucus which covers the piles, and which will prevent the proper action of the acid.—*London Lancet.*

